# Genetic imprinting analysis for alcoholism genes using variance components approach

**DOI:** 10.1186/1471-2156-6-S1-S161

**Published:** 2005-12-30

**Authors:** Sanjay Shete, Robert Yu

**Affiliations:** 1 Department of Epidemiology, Unit 1340, University of Texas M. D. Anderson Cancer Center, 1155 Hermann Pressler Boulevard, Houston, TX, 77030 USA

## Abstract

Genomic imprinting, which is also known as the parent-of-origin effect, is a mechanism that only expresses one copy of a gene pair depending upon the parental origin. Although many chromosomal regions in the human genome are likely to be imprinted, imprinting is not accounted for in the usual linkage analysis. In this study, using a variance-components approach with a quantitative phenotype ttth-FP1, we found significant evidence of imprinting at two loci, D7S1790 and D1S1631, on chromosome 1 and chromosome 7, respectively. Our results suggest that allowing for the possibility of imprinting can increase the power to detect linkage for localizing genes for alcoholism.

## Background

Genomic imprinting (also known as the parent-of-origin effect) is a mechanism by which only one copy of a gene pair is expressed, and this expression is determined by the parental origin of the copy. The deregulation of imprinted genes has been implicated in a number of human diseases. Expression of imprinted genes is regulated by allele-specific epigenetic modifications of DNA and chromatin. These modifications affect central regulatory elements that control the allele-specific expression of neighboring genes. Although many chromosomal regions in the human genome are likely to be imprinted, particularly those involved in developmental disorders, imprinting is not accounted for in the usual linkage analysis [1-8]. In this summary, we analyzed the ttth-FP1 (far frontal left side channel), a quantitative measure of alcohol dependence, using the families provided by a multi-center consortium of the Collaborative Study on the Genetics of Alcoholism (COGA) [9,10].

Alcoholism is a complex disorder with involvement of genetic and environmental risk factors. Several studies have shown familial aggregation, segregation, and linkages to several regions [11]. Therefore, the purpose of our study was to evaluate the possibility of genomic imprinting in the regions that show some evidence of linkage using a recently developed method. Several regions on chromosomes 1 and 7 have been localized using parametric and nonparametric methods of linkage and association methods that do not allow for the possibility of genomic imprinting.

## Methods

### Variance components approach

Quantitative variation in a trait often occurs because of the underlying variation in genetic factors. We recently developed a method to analyze quantitative traits using the variance components approach and allowing for imprinting as described by Shete and Amos [3] and Shete et al. [4]. Let X_i _be the phenotypic value for the *i*^th ^individual in a pedigree:



where μ is the overall mean, *g*_*i *_is the major-gene effect, *G*_*i *_is the polygenic effect, β_k _values are covariate effects that are assumed to be uncorrelated with genetic and environmental factors, and *e*_*i *_is the environmental effect. The major gene effect has a mean value of *a *when individual's genotype is BB, *d*_*1 *_when the genotype is Bb, *d*_*2 *_when the genotype bB, and -*a *when the genotype bb. Here, we assumed that the first allele is derived from the father and the second allele is derived from the mother. Let *d *be the dominance effect and *I *be the imprinting effect. Then, *d *= (*d*_1 _+ *d*_2_)/2 and *I *= (*d*_1 _- *d*_2_)/2. When *d*_1 _= *d*_2_, there is no imprinting. Shete and Amos [3] decomposed genetic variance at this locus into three parts: an additive component due to the paternally derived allele, σ^2^_af_; an additive component due to the maternally derived allele, σ^2^_am_; and the usual dominance component, σ^2^_d_. These parent-specific additive components are:  where *p *and *q *are the frequencies of alleles *B *and *b*, respectively. Also, σ^2^_af_+σ^2^_am _= σ^2^_a_.

When the imprinting coefficient *I *= 0, σ^2^_af _and σ^2^_am _are equal to σ^2^_a_/2; and, when σ^2^_af _and σ^2^_am _are equal, *I *= 0. Hence, Shete and Amos [3] proposed that a test for the equality of these two parent-specific additive variances is a test for imprinting. In an extended pedigree, one must consider an allele that is shared IBD (identical by descent) by a pair of relatives in which one of the relatives received the copy from his/her father and the other received the copy from his/her mother. So, we define "parent-specific IBD sharing between a pair of relatives *i *and *j*" asfollows:





We define π_mf,ij _and π_mm,ij _similarly. Then, the phenotypic covariance is given by [4]



From the above equation, it can be seen that the coefficients of π_*ff*,*ij*_,π_*mm*,*ij*_, and (π_*fm*,*ij *_+ π_*mf*,*ij*_) are equal if and only if σ^2^_af _and σ^2^_am _are equal, and σ^2^_af _and σ^2^_am _are equal if and only if the imprinting parameter *I *= 0 (i.e., there is no parental imprinting). Hence, the likelihood ratio test (LRT) for equality of these coefficients is a valid test for the null hypothesis of no imprinting. We do not estimate the parameters *p*, *q*, or *I *separately in the above equation, rather we estimate three parameters σ^2^_af_, σ^2^_am_, and (σ^2^_*a*_/2 - 2*pqI*^2^). Ordinarily, in a genome scan, one will test the joint null hypotheses of no linkage and no imprinting by testing σ^2^_af _= σ^2^_am _= 0.

### Distribution of the LRT

The asymptotic distribution of the LRT is complex. For testing linkage without imprinting the LRT test is assumed to be a half-and-half mixture of χ^2 ^random variable with one and zero degrees of freedom. For joint testing of linkage and imprinting, we now have three parameters in the model. The two parameters σ^2^_af _and σ^2^_am _are independent; however, the third parameter (σ^2^_*a*_/2 - 2*pqI*^2^) is correlated with the first two parameters [4]. Because this parameter was used in our model, we used a mixture of χ^2 ^distribution with 0, 1, 2, and 3 degrees of freedom with mixing parameters of 1/8, 3/8, 3/8, and 1/8 for joint testing of linkage and imprinting following the same rationale as in the standard linkage analyses using the approach of Self and Liang [12]. Similarly, for testing the linkage model without imprinting against the linkage model allowing for imprinting we used a mixture of χ^2 ^distribution with 0, 1, and 2 degrees of freedom with mixing parameters of 4/8, 3/8, and 1/8. These asymptotic distributions can be used to obtain point-wise significance of the LRT test for testing linkage and/or imprinting.

### Multipoint parent-specific IBD

Computation of multipoint parent-specific IBD is described by Shete et al. [4]. There are fouralleles at a singlelocus for the relativepair *i *and *j*. The two alleles for individual *i *are denoted by a vector (*i*_*m*_,*i*_*f*_), where *i*_*m *_and *i*_*f *_are maternal and paternal alleles, respectively. Similarly, we define the vector(*j*_*m*_, *j*_*f*_) for individual *j*. There are 15 possible ordered states of IBD between these two individuals [13]. Of these15 states, only 7 are essential for computation of IBD sharing in outbred populations. Using the notations of SIMWALK2 [14,15], we define probabilities of these states as S_9 _= (*i*_*m*_,*j*_*m*_)(*i*_*f*_,*j*_*f*_), S_10 _= (*i*_*m*_,*j*_*m*_)(*i*_*f*_)(*j*_*f*_), S_11 _= (*i*_*m*_)(*i*_*f*_,*j*_*f*_)(*j*_*m*_), S_12 _= (*i*_*m*_,*j*_*f*_)(*i*_*f*_,*j*_*m*_), S_13 _= (*i*_*m*_,*j*_*f*_)(*i*_*f*_)(*j*_*m*_), S_14 _= (*i*_*m*_)(*i*_*f*_,*j*_*m*_)(*j*_*f*_), and S_15 _= (*i*_*m*_)(*i*_*f*_)(*j*_*m*_)(*j*_*f*_). In these states, the pairs of alleles inside the parentheses are IBD. We used SIMWALK2 to obtain these 15 detailed states of identity sharing.

### Data

**Figure 1:**
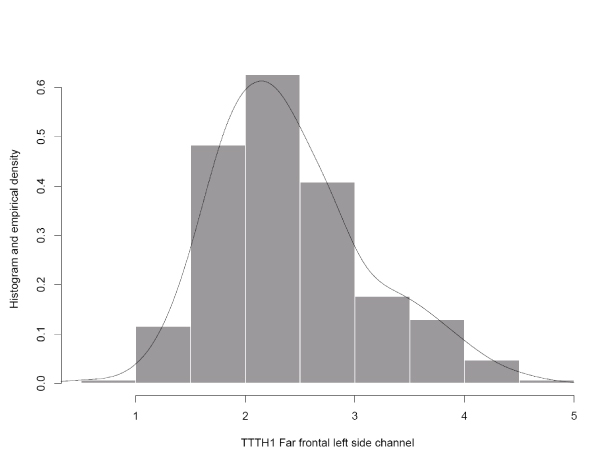
Empirical distribution of ttth1-FP1, far frontal left side channel.

To identify imprinted genes that affect the risk for alcoholism, we proposed to study 143 families consisting of a total of 1,614 individuals. We used the quantitative trait denoted by ttth1-FP1, far frontal left side channel, a quantitative measure of alcoholism. The empirical distribution of this quantitative trait is shown in the Figure 1. Because the normality of the trait distribution is underlying assumption in our variance component approach, we performed standardization of the data by subtracting mean and dividing by standard deviation followed by winsorization at a cut-off value of 1% from both side of the empirical distribution. Data winsorization has been shown to reduce the false-positive rate using variance components approach [16,17]. Finally, we used the residuals obtained from a polygenic model with sex as a covariate as our quantitative trait. From previous Genetic Analysis Workshop 11, two regions on chromosome 1 and a region chromosome 7 were selected to illustrate the utility of the method for detecting imprinting. These regions showed some evidence of linkage using standard model with at least one of the COGA defined phenotypes [11]. The markers we considered were D1S532, and D1S1631 on chromosome 1, and D7S1790 on chromosome 7 from Genetic Analysis Workshop 14 data.

## Results and Discussion

For each of the markers listed, we calculated multipoint parent-specific IBDs using the methods described. On chromosome 7, for the marker D7S1790, we obtained a negative log likelihood value of 140.35 for the model in which the major gene variance component was fixed to zero, the model without linkage. The same value under the linkage without imprinting model was found to be 135.84. Using the LRT discussed above, we obtained a suggestive significant *p*-value of 0.00129 for linkage. Furthermore, we obtained a negative log likelihood value of 129.95 using the variance components approach that tests for linkage allowing for imprinting. Using the LRT, the significance for joint testing of linkage and imprinting at this marker is 0.00003. When we compared the log likelihoods with linkage but no imprinting model with joint linkage and imprinting model, we obtain a *p*-value of 0.00057, which is significant evidence of imprinting. The evidence of imprinting is also evident from the lower *p*-value obtained using the imprinting model.

On chromosome 1, for the marker D1S1631, we obtained a negative log likelihood value of 136.90 for the model with linkage without imprinting. This gives a significance value of 0.00417 for linkage. A negative log likelihood value of 131.90 was obtained for the linked model with imprinting. This leads to a significant *p*-value of 0.00018 for joint testing of linkage and imprinting. When we compared the log likelihoods with linkage but no imprinting model with joint linkage and imprinting model, we obtain a *p*-value of 0.00143, which is a suggestive significant evidence for imprinting. Evidence of imprinting for marker D1S532 was not very significant, as shown in the Table 1. These results showed that a genome scan approach using a linkage model that allows for imprinting is important for alcoholism. There may be other regions on the genome that did not show evidence for linkage using standard models but could show significance if imprinting is allowed. Therefore, we recommend a genome scan with an imprinting model as oppose to just testing for the regions showing linkage using the standard models. Shete and Amos [3] note that it is important to allow for the sex-specific recombination fraction while testing for linkage allowing imprinting to reduce the false-positive rate.

## Conclusion

Imprinting is not accounted for traditional linkage analyses. We found evidence of imprinting, even allowing for the multiple testing, on two loci. It may also be important to allow for other covariates of environmental exposures, such as smoking, in the model. In addition, the asymptotic distribution that we used may not be very accurate and recommend simulation-based *p*-values at the significant loci to confirm evidence of linkage. In conclusion, our results suggest that allowing for imprinting in the linkage analyses can increase the power to detect genes responsible for the alcoholism.

## Abbreviations

COGA: Collaborative Study on the Genetics of Alcoholism

LRT: Likelihood ratio test

IBD: Identity by descent

## Authors' contributions

SS conceived the study, participated in the statistical analyses, and drafted the manuscript. RY participated in the statistical analyses and helped draft the manuscript.

## Figures and Tables

**Table 1:** Significance of likelihood ratio test for linkage and imprinting.

Marker	L(unlinked)	L(linked)	L(linkage and imprinting)	*p*-Value (linkage)	*p*-Value (linkage and imprinting)	*p*-Value (imprinting)
D7S1790	140.38	135.84	129.95	0.00129	0.00003	0.00057
D1S1631	140.38	136.90	131.90	0.00417	0.00018	0.00143
D1S532	140.38	137.35	134.66	0.00700	0.00270	0.01612
